# Supplementary material for: Catalyst Speciation during ansa-Zirconocene-Catalyzed Polymerization of 1-Hexene Studied by UV-vis Spectroscopy—Formation and Partial Re-Activation of Zr-Allyl Intermediates
Source: Polymers (Basel). 2019 May 29;11(6):936. doi: 10.3390/polym11060936 (PMC6630943; doi:10.3390/polym11060936)
Supplement: Supplementary file 1 [file polymers-11-00936-s001.pdf]

Catalyst Speciation During ansa-Zirconocene-Catalyzed Polymerization of 1-Hexene Studied by UV-vis Spectroscopy – Formation and Partial Re- Activation of Zr-Allyl Intermediates.

Valentina N. Panchenko [1,2], Dmitrii E. Babushkin [1], John E. Bercaw [3] *and Hans H. Brintzinger [4]

[1] Boreskov Institute of Catalysis, Russian Academy of Sciences, Siberian Branch, RU-630090 Novosibirsk, Russian Federation; panchenko@catalysis.ru, dimi@catalysis.ru
[2] Novosibirsk State University, Pirogova Str. 2, 630090, Novosibirsk, Russian Federation
[3] Arnold and Mabel Beckman Laboratories of Chemical Synthesis, California Institute of Technology, Pasadena, California 91125, USA; bercaw@caltec.edu
[4] Fachbereich Chemie, Universität Konstanz D-78464 Konstanz, Germany; hans.brintzinger@uni-konstanz.de
* Correspondence: hans.brintzinger@uni-konstanz.de

Supporting Information

Contents:

^{1}H NMR signals ascribed to species **C-2** S-1

^{1}H-NMR signals of CH_3D S-2

UV-vis spectrum of $SBIZr\text{-}CH_2SiMe_3^+$ S-3

UV-vis spectra of $SBIZr(\mu\text{-}Me)_2AlMe_2^+$ at +40°C and –20°C S-4

Plot of ln[C-0]$_t$ *vs.* t S-5

Plot of ln[C-1]$_t$ *vs. t* S-6

Effects of initial [Al]/[Zr] ratio S-7

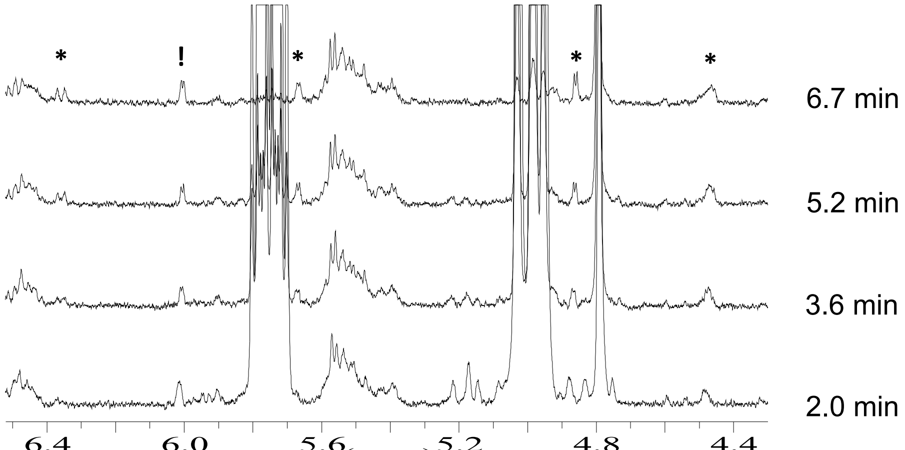

Figure S-1. ^{1}H NMR signals arising during polymerization of 1-hexene by the catalyst system described in Figure 1. First 4 traces (2.0 , 3.6, 5.2 and 6.7 min mean time). Signals at 6.36, 5.67, 4.87 and 4.48 ppm (marked by *), possibly due to polymer-carrying cations of type SBIZr-π-(1-R-2-pol-C_3H_3)$^+$ (**10**).

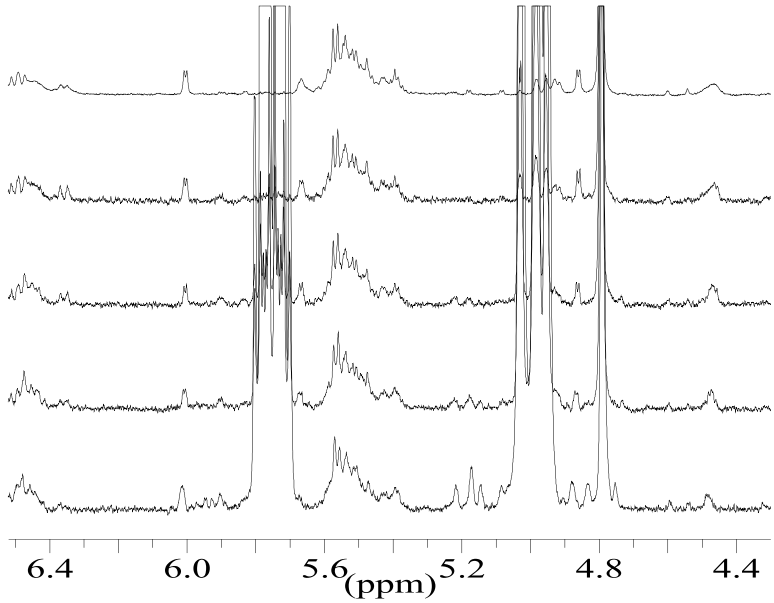

Additional traces after ca. 20 min.

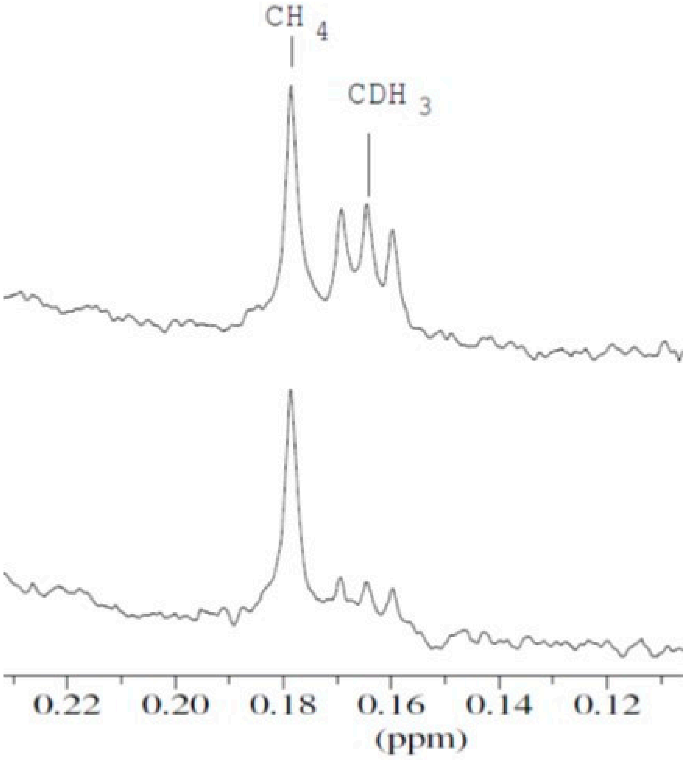

Figure S-2. ^{1}H NMR spectra of CH_4 and CH_3D in solutions obtained by addition of an equivalent amount of trityl perfluorotetraphenyl borate to a solution of SBIZrMe_2 in toluene-d_8, as described in Figure 3, after reaction times of ca. 4 h (bottom) and ca. 10 h (top).

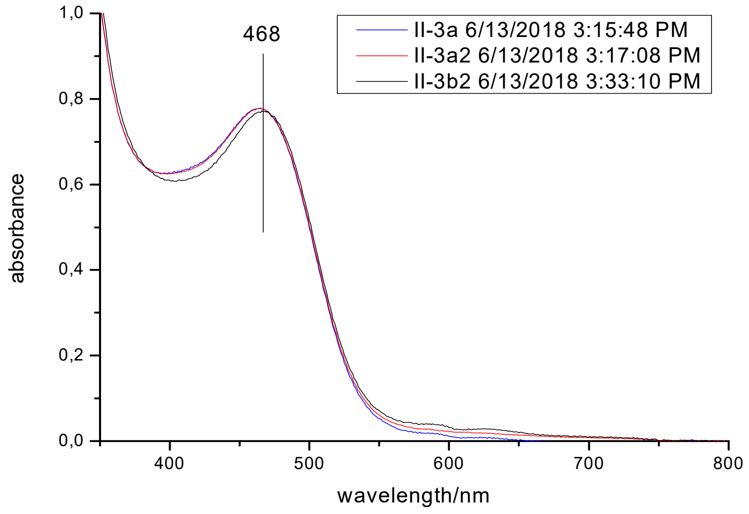

Figure S-3. UV-vis spectra of a 0.8 mM solution of SBIZr(Me)-CH_2SiMe_3 after addition of ca. 0.8 equiv. of trityl perfluorotetraphenyl borate.

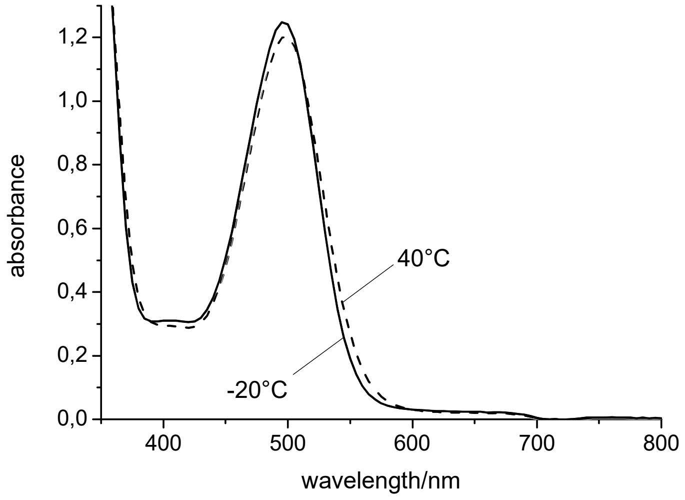

Figure S-4. UV-vis spectra of a ca. 0.5 mM toluene solution of (SBI)Zr(μ-Me)$_2$AlMe$_2^+$ B(C$_6$F$_5$)$_4^-$ at +40°C (broken line) and at – 20°C (solid line).

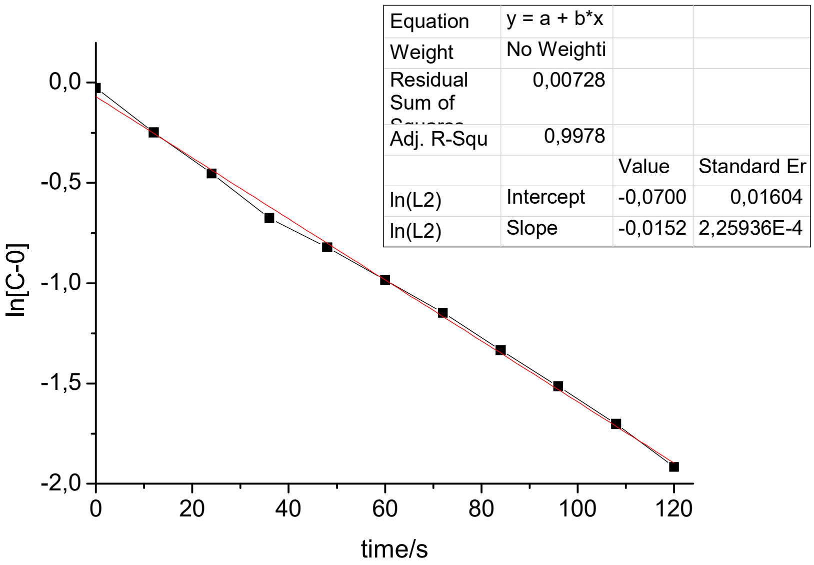

Figure S-5. Plot of ln[C-0]$_t$ vs. t for reaction stage 1 (t < 120 s):

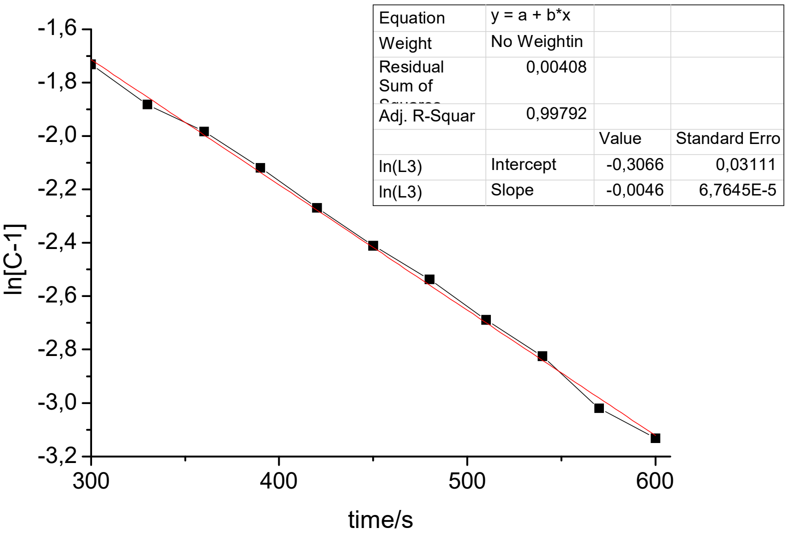

Figure S-6. Plot of ln[C-1]$_t$ vs. t for 300 s < t < 600 s

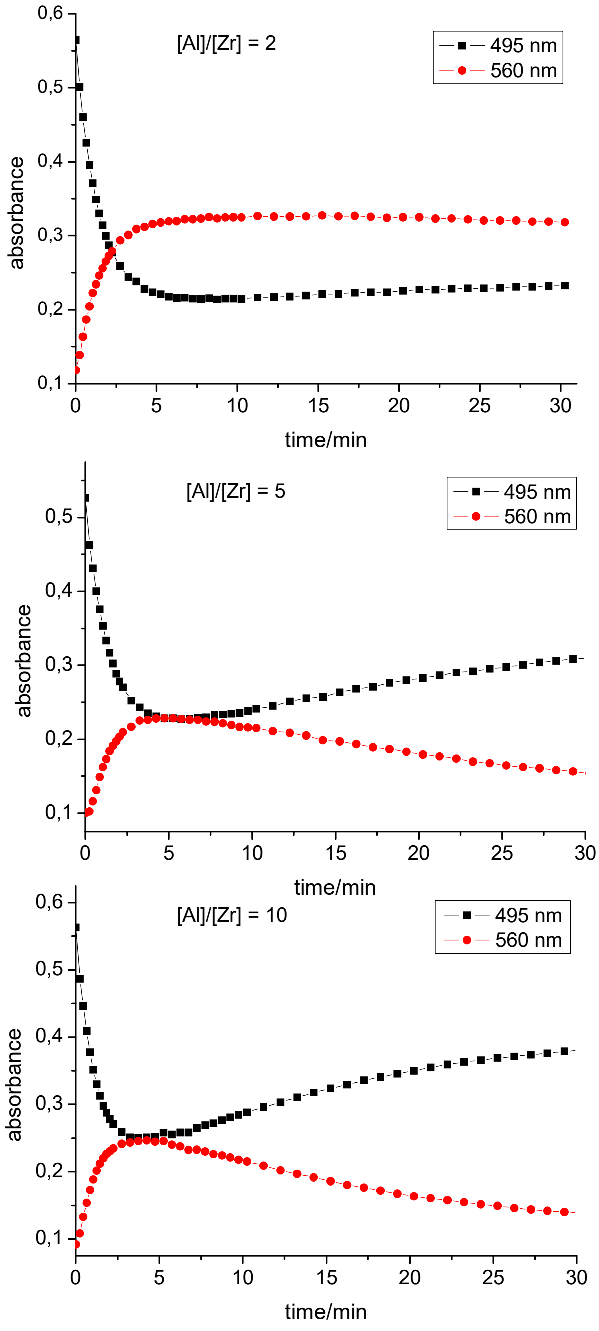

Figure S-7. Effects of initial [Al]/[Zr] ratio on the re-conversion of **C-2** (λ_{max} = 560 nm) to **C-0** (λ_{max} = 495 nm), conditions as described in Figure 1.
